# Identity, Abundance, and Reactivation Kinetics of Thermophilic Fermentative Endospores in Cold Marine Sediment and Seawater

**DOI:** 10.3389/fmicb.2017.00131

**Published:** 2017-02-06

**Authors:** Marta Volpi, Bente Aa. Lomstein, Andreas Sichert, Hans Røy, Bo B. Jørgensen, Kasper U. Kjeldsen

**Affiliations:** Center for Geomicrobiology, Department of Bioscience, Aarhus UniversityAarhus, Denmark

**Keywords:** biogeography, dipicolinic acid, dispersal, endospores, fermentative bacteria, germination, thermophiles

## Abstract

Cold marine sediments harbor endospores of fermentative and sulfate-reducing, thermophilic bacteria. These dormant populations of endospores are believed to accumulate in the seabed via passive dispersal by ocean currents followed by sedimentation from the water column. However, the magnitude of this process is poorly understood because the endospores present in seawater were so far not identified, and only the abundance of thermophilic sulfate-reducing endospores in the seabed has been quantified. We investigated the distribution of thermophilic fermentative endospores (TFEs) in water column and sediment of Aarhus Bay, Denmark, to test the role of suspended dispersal and determine the rate of endospore deposition and the endospore abundance in the sediment. We furthermore aimed to determine the time course of reactivation of the germinating TFEs. TFEs were induced to germinate and grow by incubating pasteurized sediment and water samples anaerobically at 50°C. We observed a sudden release of the endospore component dipicolinic acid immediately upon incubation suggesting fast endospore reactivation in response to heating. Volatile fatty acids (VFAs) and H_2_ began to accumulate exponentially after 3.5 h of incubation showing that reactivation was followed by a short phase of outgrowth before germinated cells began to divide. Thermophilic fermenters were mainly present in the sediment as endospores because the rate of VFA accumulation was identical in pasteurized and non-pasteurized samples. Germinating TFEs were identified taxonomically by reverse transcription, PCR amplification and sequencing of 16S rRNA. The water column and sediment shared the same phylotypes, thereby confirming the potential for seawater dispersal. The abundance of TFEs was estimated by most probable number enumeration, rates of VFA production, and released amounts of dipicolinic acid during germination. The surface sediment contained ∼10^5^–10^6^ inducible TFEs cm^-3^. TFEs thus outnumber thermophilic sulfate-reducing endospores by an order of magnitude. The abundance of cultivable TFEs decreased exponentially with sediment depth with a half-life of 350 years. We estimate that 6 × 10^9^ anaerobic thermophilic endospores are deposited on the seafloor per m^2^ per year in Aarhus Bay, and that these thermophiles represent >10% of the total endospore community in the surface sediment.

## Introduction

Cold marine sediments in Arctic and temperate regions contain dormant endospores of thermophilic anaerobic bacteria (Isaksen et al., 1994; Hubert et al., 2009, 2010; de Rezende et al., 2013; Müller et al., 2014; O’Sullivan et al., 2015). Upon laboratory incubation at 50–60°C of pasteurized sediment samples, these endospores germinate and grow to form a taxonomically diverse community of thermophilic *Firmicutes* (Müller et al., 2014) capable of mineralizing complex organic matter via fermentation and sulfate reduction (Hubert et al., 2010). The permanently low *in situ* temperatures of these environments do not support growth of thermophiles, therefore the presence of thermophilic endospores is believed to result from passive dispersal from warm source environments (Hubert et al., 2009). The identity of these habitats remains elusive. Yet, the anaerobic thermophilic endospores belong to taxonomic lineages also observed in warm ocean crust and marine subsurface oil reservoirs and it is likely that they are constantly seeping from these environments to the overlying water column (Hubert et al., 2009; Aüllo et al., 2013; de Rezende et al., 2013). A global survey showed that cold sediments from distant geographical locations connected by ocean currents share the same phylotypes of anaerobic thermophilic endospores. Although the abundance and identities of endospores present in seawater were so far not reported, this suggests that the anaerobic thermophilic endospores disperse over long distances in seawater until deposited on the seafloor (Müller et al., 2014). For this reason and since the thermophilic endospores stay dormant and therefore unaffected by environmental selection (Hanson et al., 2012) in the cold seabed, and can persist in that state for centuries (de Rezende et al., 2013), they are uniquely suited for studying routes and barriers for passive dispersal of marine microbes (Müller et al., 2014).

The detection of thermophilic anaerobic endospores in marine sediments relies upon laboratory incubations at 50–60°C in which endospores are induced to germinate and grow. By pasteurization before incubation it is possible to distinguish endospores, which survive, from vegetative cells, which are killed by pasteurization. Phylotypes that represent known thermophilic anaerobic endospore-formers are generally absent in cultivation-independent surveys of 16S rRNA gene sequence diversity in marine sediments (Müller et al., 2014). This is likely because endospores are not lysed by nucleic acid extraction procedures (Wunderlin et al., 2014) and because their abundance is low relative to vegetative microbial cells. So far, investigations of the abundance of thermophilic anaerobic endospores in cold marine sediments and estimates of their rates of supply to the seafloor only considered sulfate-reducing members of the endospore community. The abundance of thermophilic sulfate-reducing endospores has been determined in samples from surface sediments from Aarhus Bay and from Svalbard by either most probable number (MPN) enumeration (de Rezende et al., 2013) or by estimations based on measuring bulk rates of sulfate reduction in incubation experiments and relating these to cell-specific sulfate reduction rates (Hubert et al., 2009; de Rezende et al., 2016). The endospore abundances ranged from ∼10^4^ to 10^5^ endospores cm^-3^ with corresponding rates of supply of ∼10^7^–10^8^ endospores m^-2^ year^-1^ (Hubert et al., 2009; de Rezende et al., 2013, 2016). The thermophilic sulfate-reducing endospores are affiliated with the genus *Desulfotomaculum* of the *Firmicutes* family *Peptococcaceae* (Isaksen et al., 1994; Hubert et al., 2009, 2010; de Rezende et al., 2013; Müller et al., 2014; O’Sullivan et al., 2015). However, 16S rRNA gene sequencing shows that members of endospore communities are not dominated by this family, but by members of the *Firmicutes* families *Clostridiaceae* and *Bacillaceae* that are likely growing by fermentation (Müller et al., 2014). Furthermore, based on the differences in cellular growth yield between fermentative and sulfate-reducing microorganisms, fermenters should outnumber sulfate reducers several-fold in heterotrophic anaerobic microbial communities (Kirchman et al., 2014). In agreement with this, molecular surveys of anoxic marine sediments have shown sulfate reducers to make up 1–10% of the total microbial community (Lever, 2013). This suggests that thermophilic fermentative endospores (TFEs) are more abundant in the seabed than thermophilic sulfate-reducing endospores, which highlights the potential of using TFEs for tracing microbial dispersal in the marine environment.

We investigated the distribution and taxonomic identity of TFEs in the water column and sediment of Aarhus Bay (Denmark). By detecting the presence of TFEs in the water column and comparing their taxonomic identities to the identities of those present in the sediment we provided direct evidence for the role of seawater dispersal. Furthermore, we developed assays to quantify TFEs and determined their abundance in the surface and subsurface sediments. From these data we quantified their rate of deposition from the water column and their rate of survival upon burial in the sediment. Besides addressing these aims, our results show how fast environmental thermophilic endospore communities resuscitate after dormancy.

## Materials and Methods

### Sampling

Sediment samples were collected in Aarhus Bay (Denmark) at Station M5 (56°06.20 N 10°27.47 E; water depth 28 m; Langerhuus et al., 2012). Surface and bottom water temperatures in the central bay do not exceed 18 and 15°C, respectively (Jensen et al., 1988; Glud et al., 2003). Surface sediment (0–10 cm below the sea floor [cmbsf]) used in time course incubation experiments was collected in November, 2010, and January, 2016, with a box corer and immediately stored under anoxic conditions at 4°C in gas-tight plastic bags (Hansen et al., 2000). For MPN incubations, cores of undisturbed sediment were collected in January 2015 by Rumohr Lot coring (Meischner and Rumohr, 1974) and by a gravity corer. The cores were subsampled on deck using 10 mL sterile cut-off plastic syringes for collecting surface sediment (4 and 25 cmbsf) and deeper sediment (70, 120, 170, 220, 270, 370 cmbsf). The syringes were stored at 4°C in gas-tight plastic bags containing oxygen consuming packs (Oxoid^TM^ CampyGen^TM^ 2.5L) that remove O_2_ in a reaction with ascorbic acid that does not release H_2_ (Souza Cruz et al., 2012). Surface seawater (30 L) from Aarhus Bay was sampled in March 2015 from the seaside of the southern jetty of Aarhus Harbor, which reaches 2 km into the Bay, and stored at 4°C in 15 L plastic canisters.

### Preparation of Sediment Slurries

Sediment slurries were prepared by homogenizing sediment with sterile, anoxic, sulfate-free synthetic seawater medium in a 1:2 (v/v) ratio under a constant flow of sterile filtered (0.2 μm pore size) N_2_ gas. Slurries used for H_2_ measurements were prepared with argon gas in place of N_2_, facilitating detection of H_2_ production by gas chromatography. The synthetic seawater was prepared according to the saltwater basal medium recipe by Widdel and Bak (1992), omitting Na_2_SO_4_, vitamins and trace metals. The pH was adjusted to 7 with sterile 1 M NaOH or HCl solutions. Slurries were prepared with and without molybdate (Na_2_MoO_4_; 10 mM final concentration) and *Spirulina* powder (freeze-dried cyanobacterial cells, 2.5 g L^-1^ final concentration; Natur-drogeriet, Pharmax). Before addition, the *Spirulina* powder was suspended in synthetic seawater medium and sterilized by autoclaving under a N_2_ atmosphere. Slurries used for fluorescence *in situ* hybridization (FISH) analysis were prepared in a 1:9 (v/v) sediment to synthetic seawater ratio and amended with molybdate (10 mM final concentration) and *Spirulina* powder (2.5 g L^-1^ final concentration). The 1:9 dilution of these slurries facilitated the FISH analysis by reducing background fluorescence from sediment particles.

### Pasteurization of Sediment

Sediment slurries were pasteurized at 80°C in a water bath to kill vegetative cells prior to incubation. The development of the temperature inside the vials during pasteurization was determined from thermistors inserted through the butyl-rubber stoppers. The time necessary to reach 80°C was determined, as well as the time needed to cool down to 50°C at 4 or 21°C ambient temperature, or to freeze in a bath of dry ice and ethanol (Supplementary Figure S2). Slurries of 120 mL were pasteurized for 1 h while slurries of less volume were pasteurized for 40 min.

### Time Course Experiments

Anoxic sediment slurries were incubated at 50°C for monitoring germination and growth of endospores over time. Sediment slurries of 120 mL were incubated in 250 mL bottles sealed by butyl rubber stoppers under a nitrogen or argon atmosphere. Experiments were initiated by pasteurizing slurries from a starting temperature of 20°C as described above, followed by cooling to 50°C and incubation in darkness. To ensure homogeneous mixing of the slurries, the glass bottles were placed on magnetic stirrers throughout the incubation period. Slurries were sampled by disposable sterile plastic syringes via a metal needle inserted permanently through a butyl rubber stopper sealed port on the side of the glass bottles and fitted with a Luer Lock valve. Two pasteurized 120 mL slurries (replicate A and B) were incubated for 12 h at 50°C, and sub-sampled over time for concentration measurements of volatile fatty acids (VFAs) and for extraction of RNA. Parallel 120 mL slurries were sampled for concentration measurements of sulfate, H_2_ and CH_4_. Time course incubations for high-resolution measurements of DPA were performed in replicate sediment slurries of 6 mL. Aliquots from two master slurries were transferred to 75 Hungate tubes and sealed under a constant flux of sterile N_2_. One set of 43 tubes was pasteurization and placed at room temperature to cool down to 50°C and transferred into a water bath at 50°C. The remaining 32 tubes were incubated at 50°C without pasteurization. At defined time intervals, tubes were sacrificed by rapid freezing in a dry ice-ethanol bath and then stored at -20°C.

### Most Probable Number Enumeration

Most probable number enumeration of TFEs in sediment samples was performed in both natural and artificial anoxic media. All materials, reagents, and gasses utilized were steriled prior use either by autoclaving or by filtration (0.2 μm pore size). Autoclaved sediment slurries were used as natural anoxic sediment medium for further MPN incubations (Vester and Ingvorsen, 1998). The slurry was prepared in a glass bottle by diluting surface sediment 1:1 (v/v) with synthetic seawater medium. The slurry was then homogenized and passed through a 1 mm-mesh sieve. One liter of the slurry was autoclaved for 1.5 h at 120°C, allowed to cool under a N_2_ atmosphere and aliquoted (9 mL) into Hungate tubes, which were then sealed by butyl rubber stoppers under a constant stream of N_2_ gas. The tubes were incubated at 50°C for 3 days to allow residual endospores to germinate, and then autoclaved again for 1.5 h to kill these. The final pH of the slurry medium was 6.5. Prior to inoculation and use, all tubes were flushed with helium and amended with 200 μL of autoclaved *Spirulina* solution to a final concentration of 2.5 g L^-1^ and 100 μL sterile sodium molybdate solution to a final concentration of 10 mM. The artificial anoxic medium was prepared following the recipe of basal saltwater medium (Widdel and Bak, 1992), omitting Na_2_SO_4_. *Spirulina* powder and molybdate were added to the medium in final concentrations of 2.5 g L^-1^ and 10 mM, respectively. The medium was autoclaved for 30 min, cooled down under a N_2_ atmosphere, and aliquoted (9 mL) into sterile Hungate tubes which were sealed with sterile butyl rubber stoppers under a stream of N_2_ gas. The pH was adjusted to 7.4 with sterile 1M HCl or NaOH solutions and checked in randomly selected tubes upon final autoclaving. Finally, the headspace of all tubes was flushed with helium gas. For each MPN analysis, three tubes were inoculated by adding 1.0 mL of fresh sediment with a cut-off 1 mL syringe, flushing the headspace with helium gas. The inoculated tubes were mixed by vigorous shaking and pasteurized for 1 h. Ten-fold dilution series, from 10^-1^ to 10^-7^ (10^-1^ to 10^-9^ for the 4 cmbsf sample), were made in triplicates starting from the three inoculated tubes. Un-inoculated tubes (18 pcs) served as negative controls. Tubes were incubated in the dark, at 50°C, and were regularly scored for growth of fermentative bacteria by monitoring the H_2_ concentration in the headspace. Incubations were terminated when the concentration of H_2_ exceeded 1000 ppm in the headspace of the inoculated tubes. Tubes inoculated with the deepest sediment (370 cmbsf) were terminated after 7 weeks with no detectable H_2_ production in any of the dilutions. Upon termination, 3 mL samples were collected for acetate analysis, and tubes with acetate concentrations higher than 0.60 mM were scored as positive. Of the 18 negative controls performed, four showed acetate concentrations higher than 0.13 mM, with a maximum acetate concentration detected of 0.57 mM, while the maximum H_2_ concentration scored was 65 ppm.

### Seawater Filtration and Filter Incubation

Four seawater samples (4.0 L) were filtered directly through 0.2 μm pore-size Sterivex filter units (Merck Millipore) to collect planktonic cells. Subsequently, the filter units were opened in a sterile Petri dish and the filter pieces were removed with sterile forceps and transferred to 200 mL serum glass bottles containing 50 mL of (i) sterile natural anoxic sediment medium amended with *Spirulina* powder (2.5 g L^-1^) and molybdate (10 mM) as described above or (ii) sterile anoxic saltwater basal medium (Widdel and Bak, 1992) also containing 2.5 g L^-1^ *Spirulina* powder and 10 mM molybdate. The headspace of the bottles was exchanged with sterile argon gas and the bottles were sealed with butyl rubber stoppers. Subsequently, the bottles were pasteurized for 45 min as described above and then incubated in the dark at 50°C. Samples were removed by syringe and needle at regular intervals for measuring VFA and H_2_ gas concentrations and for collecting biomass for RNA extraction. All samples were preserved and processed as detailed below.

### VFA, DPA, and Sulfate Measurements

Slurry samples (3 mL) were centrifuged at 12,100 *g* for 3 min. The supernatant was filtered (0.2 μm pore size IC Acrodisc syringe Filters, PALL) into pre-combusted glass vials for concentration measurement of VFAs by either two-dimensional ion chromatography and mass spectrometry as described by Glombitza et al. (2014) or by high pressure liquid chromatography (HPLC), using a Dionex Ultimate-3000 HPLC (Thermo Fischer). HPLC separation was performed on an Aminex HPX-87H column (300 mm × 7.8 mm) under isothermal conditions (60°C) with 5 mL H_2_SO_4_ eluent (5 mM) and a flow rate of 0.6 mL min^-1^. VFAs were detected with a refractive index detector. Samples for IC analysis were diluted up to 200 times. Standards for calibration ranged in concentration from 0.75 to 6 mM, and the standard curve of each VFA followed a linear trend within this range. DPA concentrations were measured in 1 mL filtered (0.2 μm pore size) supernatants from incubated samples and from planktonic cells from 1.4 L seawater collected by filtration through a Sterivex filter cartridge (0.2 μm pore size, Merck Millipore). DPA measurement were performed according to Lomstein and Jørgensen (2012), with the following modifications: the Phenomenex Gemini C_18_ IIA column was replaced with a Waters CORTECS^®^ C_18_ (2.7 μm) 4.6 mm × 150 mm column, and the Phenomenex guard column was replaced with a CORTECS^®^ (2.7 μm) VanGuard^TM^ Pre-column 2.1 mm × 5 mm column. Furthermore, the concentration of Tb^3+^ added to standards and samples was increased from 5 to 40 μM. The concentration of DPA in each sample was calculated by use of DPA standard addition to samples, together with an un-amended sample. The conversion from DPA to endospores numbers is based on a endospore specific DPA content of 2.24 × 10^-16^ mole per endospore (Fichtel et al., 2007). For sulfate measurements 1 mL supernatants from centrifuged slurry samples were purged with CO_2_ gas to lower the pH and degas sulfide, then diluted 100 times to lower the chloride concentration. Sulfate concentration in the diluted samples was determined by ion chromatography, using the procedures and equipment described by Glombitza et al. (2014).

### H_2_ and Methane Measurements

Gas samples for H_2_ and methane concentration measurements were collected by a glass syringe with metal needle directly from the headspace of incubated slurries and preserved by injection into 10 mL butyl-rubber stopper sealed glass vials containing 1 mL saturated sodium chloride. H_2_ was measured on a Peak Performer1 gas chromatograph (GC) with reducing gas detector as described in Lin et al. (2012). Methane concentrations were measured by gas chromatography on an SRI 310C GC with flame ionization detector (SRI, Torrance, CA, USA) as described in Bárcena et al. (2010).

### Fluorescence *In situ* Hybridization

Metabolically active cells in the 1:9 (v/v) sediment slurry incubated at 50°C were quantified by FISH. Subsamples (1 mL) were fixed with 1 mL filter-sterilized (0.2 μm pore size) 1:1 (v/v) PBS (130 mM NaCl, 10 mM Na_2_PO_4_, pH 7.4)/ethanol (absolute) solution, homogenized by vortexing and stored at -20°C. Before hybridization, the fixed samples were treated by ultra-sonication to disperse microcolonies and detach cells from sediment particles as follows: 1 mL of sample was mixed with 1 mL 0.1% (w/v) sodium pyrophosphate solution in a 2 mL centrifuge tube and placed in an ice bath. The sediment slurry was then sonicated by a Sonoplus HD2070 (Bandelin) tip sonicator with a MS 72 probe for 30 s at 30% power. To allow settling of bigger sediment particles, sonicated samples were left in vertical position for 5 min before using the cleared water phase for collecting cells by filtration on to 0.2 μm pore size polycarbonate filters (Nucleopore). FISH was performed according to a standard protocol (Daims et al., 2005) with a mixture of three domain-specific probes for Bacteria (EUB338, EUB338-II and EUB338-III, Daims et al., 1999) and the non-sense probe NON (Manz et al., 1992) as control, at a formamide concentration of 35% (v/w) in the hybridization buffer. All probes were labeled with the fluorescent dye CY3. Total cell counts were performed on the same filters by DAPI (1 μg μL^-1^) staining of cells for 10 min and epifluorescence microscopy (Axiovert 200M, Zeiss).

### RNA Extraction

Samples (3 mL) used for RNA extraction were pelleted by centrifugation and frozen at -80°C immediately upon sampling. RNA was extracted with the Power Soil Total RNA Isolation Kit (Mo Bio Laboratories). Yield and purity of extracts were evaluated using a NanoDrop spectrophotometer (Thermo Scientific). RNA extracts (50 μL) were treated with 1 μL DNase (TURBO DNA-free kit, Ambion, Applied Biosystems) at 37°C for 30 min and inactivated with the Inactivation Reagent included in the kit.

### RT-PCR, PCR, and Illumina MiSeq Sequencing

Reverse transcriptase PCR (RT-PCR) amplification of 16S rRNA was performed with the OneStep RT-PCR Kit (Qiagen). Reactions of 25 μL included the buffer, dNTP and enzyme solutions supplied with the kit, 1.5 μL bovine serum albumin (BSA, 10 μg μL^-1^), 0.5 μL of each of the general bacterial primers Bac341F and Bac805R (Klindworth et al., 2013) (10 pmol μL^-1^) and 2 μL of Dnase-treated RNA template. Reverse transcription at 50°C for 30 min was followed by thermal cycling consisting of: 15 min at 95°C and 27 PCR cycles of 94°C for 40 s, 57°C for 45 s and 72°C for 1 min, plus a final elongation step of 7 min at 72°C. The PCR products were checked by agarose gel electrophoresis. As controls for the efficiency of DNase treatment, parallel PCR reactions with the non-RT-PCR-treated RNA extracts as template were performed using the Hot StarTaq Master Mix Kit (Qiagen) and the same reaction and PCR cycling conditions as for RT-PCR (the 30 min incubation at 50°C was omitted). These control-PCR reactions did not yield any product (Supplementary Figure S1).

In a second set of PCR reactions all RT-PCR products were supplied with forward and reverse Illumina adapter overhang sequences in preparation for sequencing on an Illumina Miseq system. The adaptor sequence information is provided in Illumina’s published “16S metagenomic sequencing Library preparation protocol”^1^. Reactions of 50 μL contained the following components: 25 μL 2X KAPA HiFi Hotstart Readymix (KAPA Biosystems), 1 μL of each of the primers Bac341F and Bac805R carrying the forward and reverse adapter overhang sequences at their 5′ ends (both primers at a concentration of 10 pmol μL^-1^), and 5 μL of the product from the first round of PCR as template. The thermal cycling conditions were: 95°C for 3 min, 10 PCR cycles of 94°C for 40 s, 55°C for 45 s, 72°C for 1 min, and a final elongation step of 7 min. The resultant PCR products were evaluated by agarose gel electrophoresis. Negative control PCR reactions from the first round of PCR were included in this second round and did not yield any product. PCR cleanup, indexing with the Illumina Nextera XT Index Kit, library quantification, and pooling as well as sequencing was performed following Illumina’s published 16S metagenomic sequencing Library preparation protocol. PCR products were purified with the Agencourt AMPure XP Kit (Beckman Coulter) and quantified by a Qubit 2.0 fluorometer with the dsDNA HS Assay Kit (Life Technologies) and on a 2100 Bioanalyzer system using a High Sensitivity DNA Analysis Kit (Agilent). Pooled libraries were sequenced on an Illumina MiSeq system using a 600 cycle MiSeq v3 Reagent Kit (Illumina) which produces two 300-bp long paired-end reads.

### RT-PCR, Cloning, and Sanger Sequencing

Reverse transcription and PCR amplification were carried out as described above, but with the primer combinations Bac341F (Muyzer et al., 1993) and Bac1075R (Ohkuma and Kudo, 1998) at an annealing temperature of 57°C. Parallel PCR reactions were performed to check for DNA contamination, as described above, but did not yield any product visible by agarose gel electrophoresis. PCR products were purified using the GenElute PCR Clean-Up Kit (Sigma-Aldrich) and cloned with the pGEM-T Easy Vector System (Promega) and competent cells of *Escherichia coli* strain JM 109 (Promega). Plasmid inserts were Sanger sequenced on one strand (GATC Biotech).

### Sequence Data Analyses

Forward and reverse Illumina sequencing reads were merged with the make.contigs command of the mothur program package (Schloss et al., 2009), which was also used for the downstream analyses unless otherwise noted. Sanger sequencing reads were processed along with the Illumina sequencing reads. For initial quality filtering, reads shorter than 440 nt or carrying mismatches against PCR primers or homopolymeric stretches longer than 7 nt were removed from the dataset. Reads were aligned against the SILVA SSU Ref NR 99 database (release 123, Pruesse et al., 2007). Reads aligning outside the region amplified by the PCR primers were removed from further analyses. Reads were denoised by the pre.cluster command and screened for chimeric sequences by the chimera.uchime algorithm (Edgar et al., 2011) implemented in mothur. Reads were clustered into operational taxonomic units (OTUs) with the pick_otus.py command in Qiime (Caporaso et al., 2010). Using a custom Perl script, the resultant Qiime output file was converted to a .list file format compatible with further processing in mothur. Singleton OTUs were removed from further analyses and the remaining OTUs were taxonomically classified based on the SILVA SSU Reference Taxonomy (release 123). Representative sequences of each of the 2,447 OTUs detected (obtained by the get.oturep command of the mothur program package) were deposited in GenBank under accession numbers KX955270 to KX957713. The distribution of OTUs among the individual sequence libraries and their relative abundances are shown in Supplementary Table S2.

## Results

### Fermentative Activity of Germinating TFEs in Time Course Incubations

**FIGURE 1:**
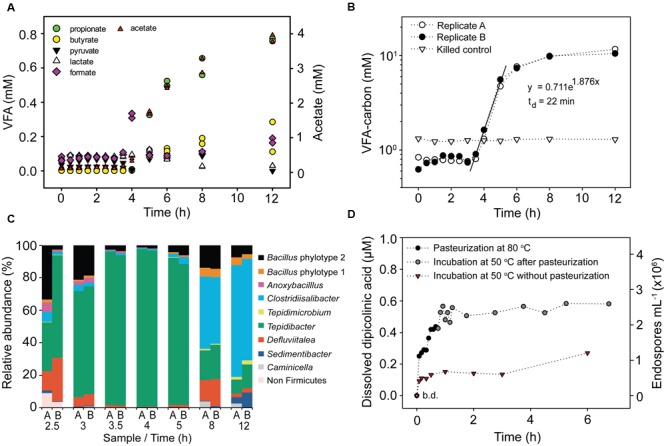
**Activity of germinating endospores in anoxic incubation experiments with pasteurized slurries of surface sediment amended with 2.5 g L^-1^*Spirulina* extract and 10 mM molybdate. (A)** Concentration of volatile fatty acids (VFAs) during incubation at 50°C of two replicate slurries. The concentration of acetate is shown on a separate scale. **(B)** Cumulated concentration of VFA-carbon during incubation at 50°C. VFA-carbon was calculated by multiplying the molar amount of the VFAs shown in **(A)** by the number of carbon atoms they contain. The concentration is shown on a log scale. **(C)** Taxonomic profiles of germinating endospores during incubation at 50°C based on Illumina sequencing of 16S rRNA cDNA. Only genera with abundance > 2% in sequence libraries are shown. 16S rRNA was not detectable by RT-PCR after 0.5, 1.0, 1.5, or 2.0 h of incubation (Supplementary Figure S1). **(D)** Concentration of dissolved dipicolinic acid (DPA) and the corresponding endospore abundance during pasteurization and incubation at 50°C.

To monitor germination and growth of TFEs we pasteurized surface sediment slurries amended with *Spirulina* powder and molybdate and measured the accumulation of VFAs during anoxic incubation at 50°C. The pasteurization was performed for 1 h, during which slurries had reached a uniform temperature of 80°C after 25 min (Supplementary Figure S2A). The pasteurization therefore included >30 min exposure to 80°C, which effectively kills mesophilic vegetative cells (Moerman et al., 2001). After pasteurization, 20–25 min was required for slurries to cool to 50°C when placed at 4°C (Supplementary Figure S2A). Time zero was defined where 50°C was reached. VFA concentrations began to increase after 3.5 h of incubation at 50°C, with acetate accumulating to 5 to 10-fold higher concentrations than the other VFAs measured (**Figure 1A**). We therefore used acetate as a proxy for VFA production in the incubation experiments. The total VFA concentration initially increased exponentially for 1.5 h after which the rate of production leveled off (**Figure 1B**). This timing and pattern of detectable VFA production was highly reproducible across replicate incubations of surface sediment (**Figure 1B**, Supplementary Figures S3A, S4, and S6). The same timing and pattern was observed for H_2_ production (Supplementary Figure S4B). The initial thermophilic production of acetate was stimulated by the presence of *Spirulina* powder (Supplementary Figure S3A), and was identical for pasteurized and non-pasteurized sediment slurries (Supplementary Figure S4A). The presence or absence of molybdate did not affect the initial production of acetate (Supplementary Figure S3B), but molybdate effectively inhibited sulfate consumption. However, even in absence of molybdate sulfate consumption was not detectable until after 20 h of incubation (Supplementary Figure S3B). Methane production was not detected in any incubation.

We also investigated the presence and activity of TFEs in seawater by collecting planktonic cells on membrane filters, followed by pasteurization and anoxic incubation at 50°C in both natural autoclaved sediment medium and artificial medium to provide diverse growth conditions and thereby maximize the detection of TFEs. Acetate production was observed in all incubations, yet at a slower rate than in surface sediment slurries (Supplementary Figure S5).

### Timing of Endospore Germination

The time of TFEs germination was determined by monitoring the concentration of dissolved DPA during pasteurization and anoxic incubation at 50°C of surface sediment slurries. DPA is an abundant molecule in the core of endospores and during the germination process it is released and replaced by water (Setlow, 2003). The experiment was performed with 6 mL sediment slurries in Hungate tubes. These were heated to 80°C in less than 2 min during pasteurization and cooled from 80°C to the incubation temperature of 50°C in 4 min (Supplementary Figure S2). Dissolved DPA was not detectable prior to pasteurization (**Figure 1D**). However, DPA accumulation was observed after only 5 min of pasteurization and DPA continued to accumulate over the whole duration of the pasteurization (**Figure 1D**). When the pasteurized slurries were moved to 50°C, a further DPA release was observed during the first 30 min of incubation after which the dissolved DPA concentration showed little further change (**Figure 1D**). In slurries incubated at 50°C without prior pasteurization DPA was released within the first 5 min of incubation, and reached 30–50% of the amounts released in the pasteurized slurries (**Figure 1D**). Based on the measured concentrations of dissolved DPA we estimate that 0.7 × 10^6^ and 2.6 × 10^6^ endospores mL^-1^ germinated in the non-pasteurized and the pasteurized slurries, respectively (**Figure 1D**).

### Abundance of TFEs in the Sediment

**FIGURE 2:**
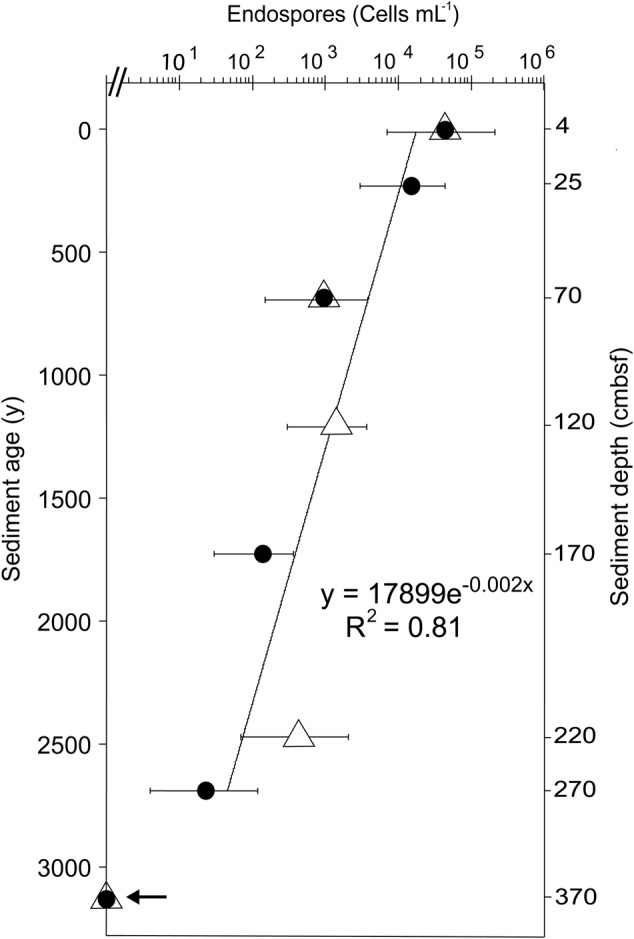
**Depth distribution of TFEs in Aarhus Bay sediment as determined by MPN enumeration in natural medium (filled circles) and artificial medium (open triangles).** Error bars show 95% confidence intervals of MPN counts. Sediment age was determined by ^14^C-dating of bivalve shells (Langerhuus et al., 2012). The line shows the exponential fit of the decrease in TFE abundance with sediment age. Growth was not detected in samples from 370 cmbsf depth, as indicated by an arrow.

The depth distribution of cultivable TFEs was determined by MPN enumeration using both natural sediment medium and artificial medium for the incubations. Triplicate 10-fold serial dilutions were performed to statistically estimate the number of TFEs in pasteurized sediment samples from eight different sediment depths. With both types of media, we detected an average number of 4.3 × 10^4^ TFEs mL^-1^ at the sediment surface with 95% confidence limits ranging from 7 × 10^3^ to 2.1 × 10^5^ TFEs mL^-1^ (**Figure 2**). The number of TFEs decreased exponentially with depth to 23 TFEs mL^-1^ at 270 cmbsf (**Figure 2**). Growth was not detected in any of the MPN dilutions of sediment samples from 370 cmbsf.

We estimated the abundance of TFEs in surface sediment from the rate of VFA production in anoxic sediment slurries incubated at 50°C (**Figure 1B**). We fitted an exponential function to the increase in the mean VFA concentration between 3.5 and 5 h of incubation of the two replicate slurries and used the mean of the VFA concentrations measured between 0 and 3.5 h as the starting point for the fit (**Figure 1B**). We assume that the exponential increase in VFA concentration was caused by exponential growth of VFA-producing populations. The rate of increase of VFA concentration for a given time point between 3.5 and 5 can be calculated by differentiating the fitted exponential function. The calculated initial rate of increase during the exponential phase (3.5 h since the beginning of the incubation) was 1.3 mmol VFA-carbon L^-1^ h^-1^. The number of germinated endospores was calculated by dividing this rate with the average cell-specific rate of VFA-carbon production. The latter rate was determined empirically in a parallel incubation experiment, dividing the VFA-carbon production rate by the number of cells detectable by FISH at a given time point (Supplementary Figure S6). No FISH signal was detected at the beginning of the incubation, demonstrating that the pasteurization effectively killed vegetative cells and that only cells of germinated and growing endospore populations subsequently produced a FISH signal. We observed a cell-specific rate of 640 fmol VFA-carbon cell^-1^ h^-1^. A comparable rate was observed in early exponential phase cultures of the mesophilic fermenter *Clostridium sporogenes* with an average cell specific rate of glucose fermentation of 100 fmol glucose-carbon consumed cell^-1^ h^-1^ [calculated from data in Lovitt et al., 1987 assuming a cellular dry weight of 0.7 pg cell^-1^ (1 pg = 10^-12^ g); Lynch and Marinov, 2015]. From the observed rate we estimated that the two replicate sediment slurries shown in **Figure 1B** contained 2 × 10^6^ TFEs mL^-1^, i.e., TFEs which germinated and grew. Since the slurry consisted of two parts of saltwater medium and one part of sediment, this number converts to an abundance of TFEs in surface sediment of 6× 10^6^ mL^-1^. The robustness of this approach can be exemplified by considering the initial rate of VFA-carbon production measured in a slurry consisting of 1 part sediment and 9 parts medium. The 1:9 slurry is 3.3 fold more diluted than the 1:2 slurry shown in **Figure 1B**, and accordingly the initial rate of VFA-carbon production differed 3.9 fold between the two slurries (Supplementary Figure S6).

### Taxonomic Identity of TFEs in Sediment and Seawater

For both replicate sediment slurries shown in **Figure 1B**, total RNA was extracted and the community composition of active bacteria was profiled by RT-PCR and Illumina sequencing of 16S rRNA. The two replicate incubations produced highly similar results (**Figure 1C**). 16S rRNA was not detectable by RT-PCR until after 2.5 h of incubation, with samples from 0.5, 1.0, 1.5, or 2.0 h of incubation producing negative results (Supplementary Figure S1). For each remaining time point, >99% of the community was composed of bacteria from the phylum *Firmicutes*. The community composition changed over time during the incubation, from an initial predominance of *Tepidibacter* (2.5–5 h), to predominance of *Clostridiisalibacter* at 8 h (**Figure 1C**).

**FIGURE 3:**
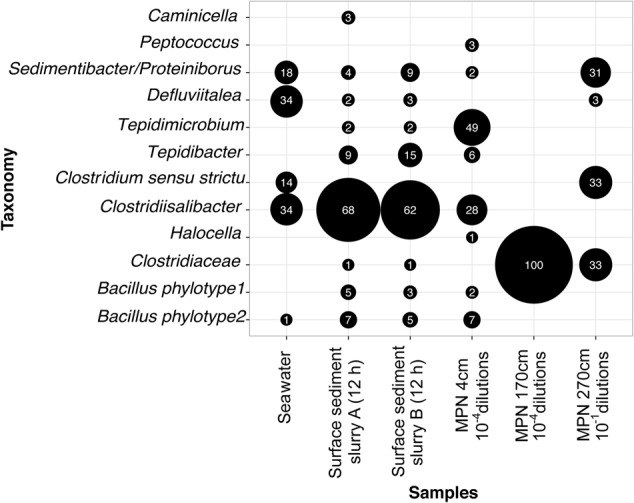
**Genus composition of germinating and growing TFEs in anoxic incubations of pasteurized planktonic cells from seawater sampled after 17 h of incubation, pasteurized surface sediment slurries sampled after 12 h of incubation (shown in **Figure 1**) and the outer-most MPN dilutions of sediment from three different depths positive for growth.** For the seawater sample pooled data from three independent incubations are shown (Supplementary Figure S5). The size of the black bubbles is proportional to the relative abundance of genera in the individual sequence datasets. Numbers inside bubbles indicate relative abundance in percent. Only genera with abundance > 2% in at least one of the datasets are shown. The sequence data were derived by cloning-Sanger sequencing (Seawater, MPN 170 cm, MPN 270 cm) or by Illumina sequencing (Surface sediment, MPN 4 cm).

**FIGURE 4:**
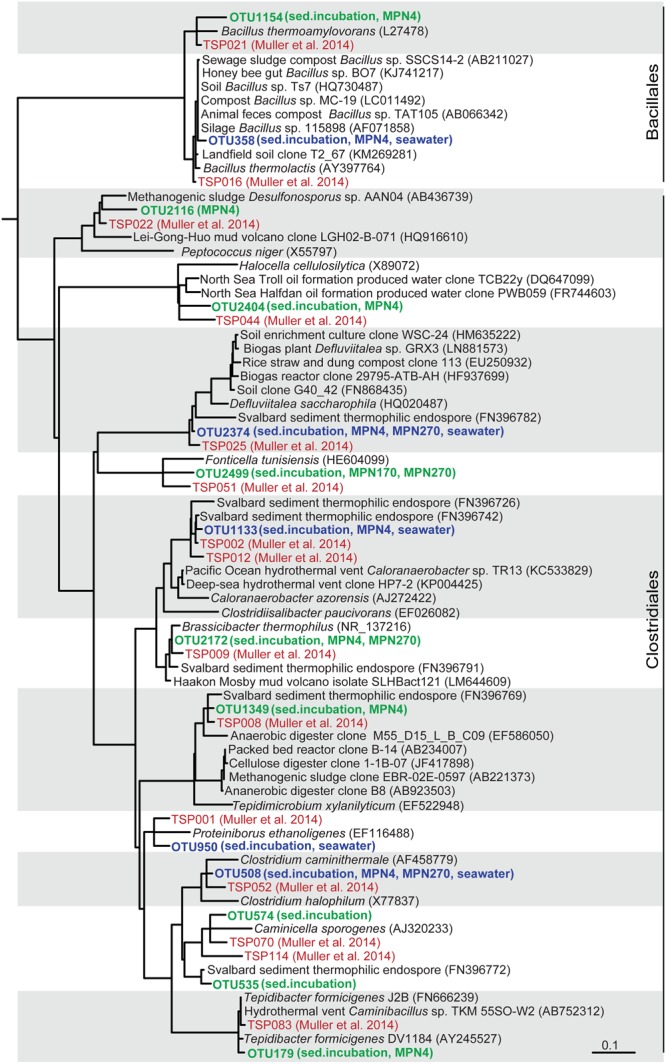
**Tree showing the phylogenetic affiliation of TFE 16S rRNA sequence OTUs derived from seawater and sediment slurry incubations and from the outermost MPN dilutions, positive for growth, of sediment from 4 cm, 170 cm, and 270 cm depth (shown in bold green).** TFE OTUs shared among sediment and seawater are shown in bold blue. Sequences from a global survey of the identity of thermophilic endospores in marine sediments (Müller et al., 2014) are shown in red color. TFE sequences derived from a previous study of Svalbard sediment (Hubert et al., 2009) are also included in the tree. The tree is based on the pre-computed guide tree of the Silva Living Tree Project ARB database release 123 (Munoz et al., 2011). Sequences from the present study and reference sequences were added to the tree without changing the overall tree topology with the parsimony tool of the ARB program package (Ludwig et al., 2004). Sequences from the Müller et al. (2014) study are identified by their OTU number (TSPXXX, where X refers to a digit). Other reference sequences are identified by their GenBank accession numbers. The scale bar shows 10% estimated sequence divergence.

To identify the most abundant types of TFEs in the sediment we RT-PCR amplified and sequenced 16S rRNA from the outermost positive dilutions of the MPN incubations. The RT-PCR products from the pooled 10^-4^ MPN triplicate dilutions of surface sediment was profiled by Illumina sequencing, while the products from 170 (single 10^-4^ dilution) and 270 (pooled triplicate 10^-1^ dilutions) cmbsf were profiled by cloning and Sanger sequencing. Different clostridial phylotypes dominated the MPN dilutions from the individual sediment depths, and represented a subset of the phylotypes observed in the sediment slurries (**Figures 3** and **4**).

Similarly, we also identified TFEs germinating and growing in the incubations of pasteurized planktonic cells from seawater (Supplementary Figure S5). 16S rRNA was RT-PCR amplified from RNA extracted from samples taken after 17 h incubation in both natural and artificial medium followed by cloning and Sanger sequencing of the amplification products (Supplementary Figure S5B). No 16S rRNA was detectable by RT-PCR at the beginning of the incubations. All sequences were either *Clostridia* or *Bacilli* and represented the same phylotypes as identified in the sediment incubations (**Figures 3** and **4**).

## Discussion

### Germination and Abundance of Endospores

We used a cultivation-dependent approach for quantifying and identifying TFEs present in our samples. Cultivation allows unambiguous discrimination of endospores from vegetative cells and thermophilic endospores from psychro- and mesophilic endospores. Furthermore, it circumvents the need for endospore permeabilization and lysis, which challenge the use of molecular approaches for studies of endospore populations in environmental samples (de Rezende et al., 2016). Our approach, however, depends on the germination and growth of the endospores, which may lead to a minimum-estimate of endospore abundance.

Endospores germinate by an ordered multistep process that is known from pure culture studies of *Bacillus* and *Clostridium* isolates (Paredes-Sabja et al., 2011; Setlow, 2014). The process is induced when specific germinant molecules activate germinant receptors in the endospore envelope, which leads to the release of cations and DPA into solution through channels localized in the envelope. This is followed by the hydration of the endospore core and the degradation of its cortex and outer layers, with a concomitant successive reactivation of metabolic processes (Sinai et al., 2015). Finally, an outgrowth phase begins where endospores transform into vegetative cells and synthesize macromolecules in preparation for the first cell division.

In *Bacillus subtilis* and in *Clostridium difficile* the full germination process lasts around 3 h with the outgrowth phase beginning after 1 h (Dembek et al., 2013; Sinai et al., 2015). We observed a similar time course for the germination of TFEs from Aarhus Bay surface sediment. DPA was released during pasteurization and during the initial 30 min of incubation of sediment slurries at 50°C, and the concentration of dissolved DPA showed little further increase thereafter (**Figure 1D**). This suggests that the majority of TFEs germinated more or less simultaneously from the beginning of the incubations. In agreement, all predominant TFE endospore OTUs detected by the 16S rRNA community profiling of the slurry incubations appeared within the initial 3 h of incubation (Supplementary Figure S7). After 3.5 h of incubation, fermentation products began to accumulate exponentially suggesting the onset of cell division and thus the end of the outgrowth phase of germinated TFEs (**Figures 1A,B**).

The amount of DPA released upon incubation at 50°C of pasteurized sediment slurries approximately match the amount released in non-pasteurized sediment slurries which correspond to 0.7 × 10^6^ endospores mL^-1^ slurry (**Figure 1D**). This suggests that the pasteurization caused non-thermophilic endospores to release their DPA possibly killing them in the process. The latter would explain why de Rezende et al. (2013) observed low activity of germinating sulfate-reducing endospores in pasteurized marine sediment samples at mesophilic as compared to thermophilic incubation temperatures. The heat resistance of endospores is species-specific, being <80°C for some species (Grecz and Tang, 1970; Warth, 1978) and when killed by heat some endospores are known to release their DPA while others are not (Kort et al., 2005; Coleman et al., 2007). However, endospores reportedly germinate at temperatures reflecting the temperature range of growth of the vegetative cells (Levinson and Hyatt, 1970; Foerster, 1983) and they can tolerate exposure to 80°C for prolonged periods without releasing their DPA (Fairhead et al., 1993). Our finding of a possible substantial DPA release from non-thermophilic endospores during pasteurization is therefore surprising. Heating at sub-lethal temperature can potentiate and synchronize endospore germination by sensitizing germinant receptors to activation by germinants (Setlow, 2014). A diverse range of such germinants is likely present in the *Spirulina* powder utilized as organic substrate as its absence caused fewer TFEs to germinate (Supplementary Figure S3A). It is possible that such potentiation may also contribute to the difference in DPA release between the pasteurized and the non-pasteurized slurries (**Figure 1D**).

*Bacillus* endospores rely on endogenous energy and carbon for fueling the initial part of the germination process while the endospores are still highly impermeable. They then switch to a regular aerobic metabolism and depend on exogenous energy sources as the outgrowth phase begins (Setlow and Kornberg, 1970; Sinai et al., 2015). Little is known about the metabolic processes fueling the germination and outgrowth of anaerobic fermentative endospores, but such endospores expectedly rely on generating ATP by fermentation of exogenous substrates during outgrowth. Considering that 10^6^ TFEs mL^-1^ slurry germinated, their catabolism would produce 2.5 mM VFA-carbon during a 2 h outgrowth phase, provided that their average cell specific VFA-carbon production rate matched that determined for growing vegetative cells. We did not observe accumulation of fermentation products in sediment slurries until 3.5 h after the germination of endospores (**Figures 1A,B**, and Supplementary Figure S4). The germinating TFEs therefore likely catabolized at much lower rates than vegetative cells until very late in the germination process.

Supporting this interpretation, the activities of glycolytic and fermentative enzymes were found to be very low in germinating spores of *Clostridium botulinum* as compared to vegetative cells (Simmons and Costilow, 1962) and genes encoding functions involved in sugar uptake were down-regulated in germinating *C. difficile* spores (Dembek et al., 2013). The abrupt exponential increase in VFA and H_2_ concentrations observed in incubated slurries can best be explained by the onset of cell division upon outgrowth. Notably, this increase cannot be explained by up-regulation of fermentative activity during the outgrowth of germinated TFEs because this would demand an unrealistically high abundance of TFEs. From the initial rate of VFA-carbon production (**Figure 1B**) we estimated slurries to contain 2 × 10^6^ TFEs mL^-1^ corresponding to 6 × 10^6^ TFEs mL^-1^ sediment, which agrees with the estimate based on DPA release (**Figure 1D**).

From the exponential increase in VFA-carbon concentration we estimated a doubling time of the germinating endospore populations of 22 min (**Figure 1B**). Such extremely short doubling times are not unusual for fermentative clostridia. For example *Tepidibacter formicigenes* has a doubling time of 16 min (Urios et al., 2004) and the doubling time of *Clostridium perfringens* is as low as 6.3 min (Labbe and Huang, 1995). Notably, *Tepidibacter* was dominating the 16S rRNA sequence libraries of the slurry incubations (**Figure 1C**) and the most abundant *Tepidibacter* phylotype (OTU179) shares 99% sequence identity with the 16S rRNA gene sequence of the fast growing *T. formicigenes* (**Figure 4**).

In conclusion the time course incubation experiment shows that our incubation procedure was highly efficient in reactivating dormant TFEs from surface layers of cold marine sediments. Our data suggest that TFEs are highly abundant, as our estimates of 6 × 10^6^ TFEs mL^-1^ sediment make TFEs 100-fold more abundant than endospores of thermophilic sulfate reducing bacteria (de Rezende et al., 2013, 2016). This estimate of TFE abundance in the surface sediment is higher than the estimate obtained by MPN of 7 × 10^3^ to 2 × 10^5^ TFEs mL^-1^ (**Figure 2**). Due to attachment to sediment particles, cultivation bias and inability to score low levels of metabolic activity, MPN methods may however, underestimate cell abundances. For example, depending on the incubation medium MPN counts of sulfate-reducing bacteria in sediment samples may vary up to 1000-fold (Vester and Ingvorsen, 1998). Thus our MPN enumerations likely represent underestimates, while the slurry-based enumeration better reflects the true TFE abundance.

### Taxonomic Identity of TFEs

We investigated the community composition of germinating TFEs during incubation at 50°C by RT-PCR amplification and sequencing of 16S rRNA. The replicate incubations were initially dominated by the genera *Tepidibacter, Defluvitalea* and *Bacillus*, and at later time points by *Clostridiisalibacter* (**Figures 1C** and **4**). The *Tepidibacter, Clostridiisalibacter* and *Bacillus* OTUs were also detected in the outermost MPN dilutions of surface sediment positive for growth, which shows that they represent predominant members of the endospore community (**Figures 3** and **4**). These OTUs, as well as several other less abundant OTUs, were closely related to globally distributed anaerobic thermophilic endospore phylotypes identified by Müller et al. (2014) (**Figure 4**). Most OTUs of high abundance in the surface sediment were not detected in MPN dilutions of samples from the deeper part of the sediment, which were characterized by lower diversity (**Figure 3**). This indicates that the rate of survival or loss of culturability during burial is species-specific.

For the first time, we detected anaerobic thermophilic endospores in seawater and determined their taxonomic identity (Supplementary Figure S5). All phylotypes enriched from seawater were also detected in the sediment (**Figures 3** and **4**), showing that the thermophilic endospores in the sediment could well-originate from the seawater.

Several of the abundant and globally dispersed (Müller et al., 2014) TFE OTUs identified in Aarhus Bay were closely related to characterized isolates all representing strict or facultative anaerobic endospore-forming species (**Figure 4**; Supplementary Table S1). Common to the characterized isolates closely related to the TFE OTUs is that they are true thermophiles that are unable to grow at temperatures below 25–40°C. This supports the assumption that the thermophilic endospores are unable to germinate and proliferate in the sediments of Aarhus Bay (de Rezende et al., 2013) and that the endospores are not formed *in situ* in the sediments by a vegetative community. Some of them, including the TFE OTU of greatest abundance in the surface sediment, *Tepidimicrobium* OTU 1349, were related to isolates and environmental sequences from terrestrial sources, which indicates that some TFEs may be of terrestrial rather than marine origin (Supplementary Table S1). These TFEs may be dispersed to the sea via rivers or by aerosolization and atmospheric transport, as was suggested as a main dispersal mechanism for aerobic thermophilic *Geobacillus* endospores (Zeigler, 2014). Other OTUs were, however, closely related to isolates and sequences originating from marine hydrothermal vents, oil reservoirs and geothermal springs (Supplementary Table S1), substantiating the hypothesis that marine TFEs originate from the warm marine subsurface and are dispersed by ocean currents (Hubert et al., 2009; de Rezende et al., 2013).

### Rate of Deposition of TFEs and Their Persistence in Sediments

Our quantification of TFEs in the surface sediment allows us to refine previous estimates of the rate of deposition of anaerobic thermophilic endospores from the water column (Hubert et al., 2009; de Rezende et al., 2013). Given a TFE abundance of 6.0 × 10^6^ mL^-1^ sediment, a sulfate-reducing endospore abundance of 10^4^ mL^-1^ (de Rezende et al., 2013), and a sedimentation rate of 0.09 cm year^-1^ (Flury et al., 2016), 5.8 × 10^9^ endospores m^-2^ year^-1^ were deposited over the past 100 years. Aarhus Bay is a depositional basin with a mean water depth of 15 m and a mean water residence time of 12 days (Jørgensen, 1996; Jensen and Bennike, 2009). Provided a rate of deposition of 5.8 × 10^9^ thermophilic anaerobic endospores m^-2^ sediment year^-1^ in Aarhus Bay, the seawater that pass through the bay must deliver around 12 thermophilic anaerobic endospores from each mL of water that enters. This rate of supply from the water column is viable, as 12 endospores mL^-1^ likely only constitute a small proportion of the endospores that are suspended in the water column of Aarhus Bay: we quantified total planktonic endospores by DPA measurements and found 200 endospores mL^-1^, and Nielsen et al., (accepted) used microcalorimetry to estimate that the water column of Aarhus Bay contains as many as 1000 thermophilic anaerobic endospores mL^-1^.

According to MPN enumeration, the abundance of culturable TFEs decreases exponentially with sediment depth and age (**Figure 2**). From the exponential fit to the decrease in TFE abundance with sediment age, an average half-life of 350 years for this endospore community can be estimated, assuming that there is no autochthonous production of TFEs in the sediment. This half-life is very similar to the half-life of culturable endospores of thermophilic sulfate-reducing bacteria (330 years) present in the same sediment (de Rezende et al., 2013). This suggest an inherent control on the loss of viability or culturability of endospores over time such as decay of nucleic acids or inactivation of enzymes or germinant receptors (Nicholson et al., 2000; Willerslev et al., 2004; Johnson et al., 2007; Ghosh et al., 2012). Another mechanism that may contribute to the decrease in thermophilic endospore abundance in marine sediment is spontaneous germination (Epstein, 2009; Buerger et al., 2012), followed by death of the newly activated vegetative cell, because germinated endospores cannot grow or survive in this cold environment.

## Conclusion

Based on quantification of DPA, the surface sediment of Aarhus Bay was previously reported to contain ∼10^7^ endospores mL^-1^ (Langerhuus et al., 2012). Our analyses of TFE abundances suggest that thermophiles make up a large proportion (>10%) of the total endospore community as a result of a high rate of deposition from the water column. Dormant TFEs germinate within minutes and resuscitate within a few hours of incubation at 50°C, and can be readily detected and identified in both sediment and seawater. Additionally, different TFE phylotypes appear to have different source environments, marine or terrestrial. These characteristics make TFEs ideally suited for studying pathways and barriers of microbial dispersal in the marine environment.

## Author Contributions

MV, BBJ, and KUK designed the study with input from HR. MV performed the experimental work, except DPA measurements which were performed by BAaL, and seawater incubations which were performed by AS. MV and KUK analyzed data and wrote the manuscript with input from BAaL, BBJ, and HR.

## Conflict of Interest Statement

The authors declare that the research was conducted in the absence of any commercial or financial relationships that could be construed as a potential conflict of interest.

## Footnotes

1 http://support.illumina.com
