# Supplementary material for: Identity, Abundance, and Reactivation Kinetics of Thermophilic Fermentative Endospores in Cold Marine Sediment and Seawater
Source: Front Microbiol. 2017 Feb 6;8:131. doi: 10.3389/fmicb.2017.00131 (PMC5292427; doi:10.3389/fmicb.2017.00131)
Supplement: Supplementary file 2 [file Presentation_1.PDF]

Supplementary Material

Identity, abundance and reactivation kinetics of thermophilic fermentative endospores in cold marine sediment and seawater

Marta Volpi, Bente Aagaard Lomstein, Andreas Sichert, Hans Røy, Bo Barker Jørgensen, Kasper Urup Kjeldsen*

*** Correspondence:**
Kasper Urup Kjeldsen, Center for Geomicrobiology, Department of Bioscience, Aarhus University, DK-8000 Aarhus, Denmark
kasperuk@bios.au.dk

Supplementary Figures and Tables

Supplementary Figures

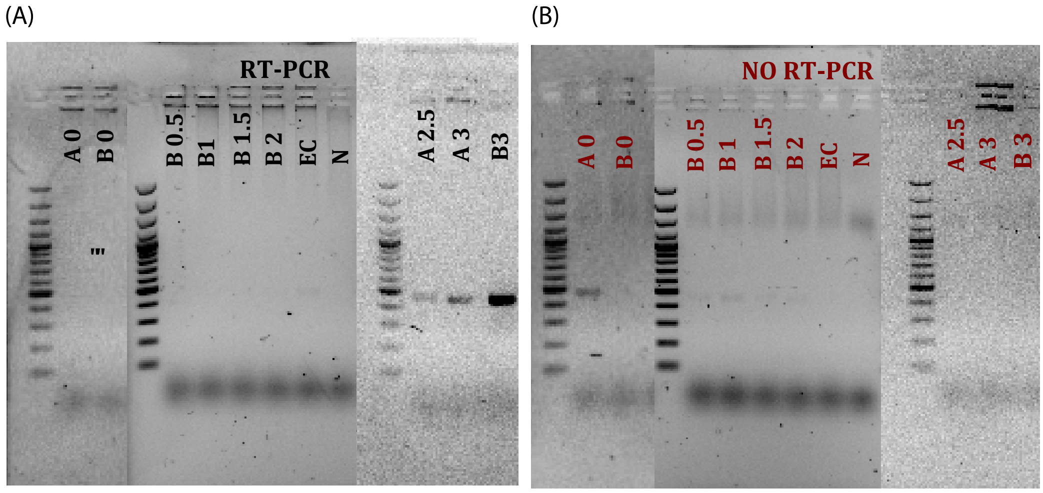

Supplementary Figure S1. Negative images of agarose gels with 16S rRNA RT-PCR products **(A)** and products from parallel control PCR for DNA contamination **(B),** amplified from DNase treated RNA extracts from pasteurized surface sediment slurries incubated at 50°C (see Figure 1B). A0 and B0 = replicate slurries A and B at time zero of their incubation. B0.5 to B2= replicate B each half an hour for the first 2 hours of incubation, EC = RNA extraction negative control, N = (RT)PCR blank.

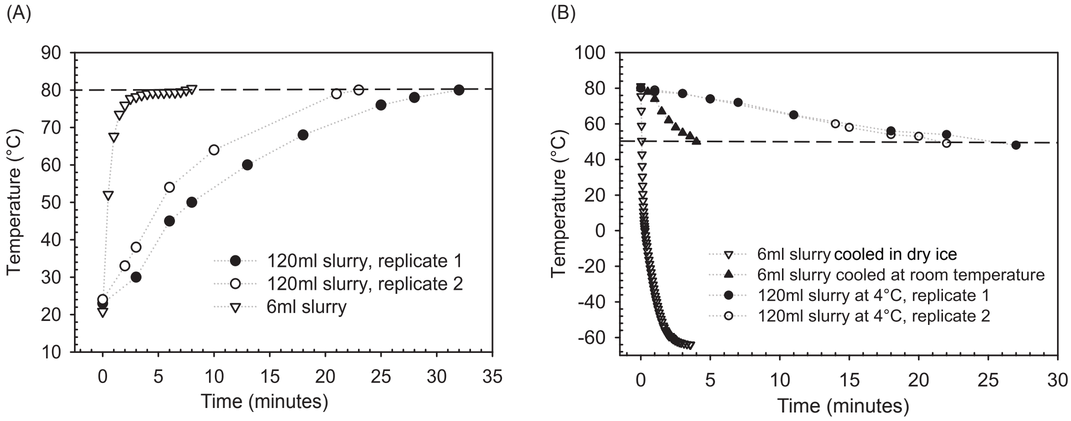

Supplementary Figure S2. Temperature equilibration of sediment slurries during **(A)** pasteurization at 80°C and **(B)** cooling upon the pasteurization.

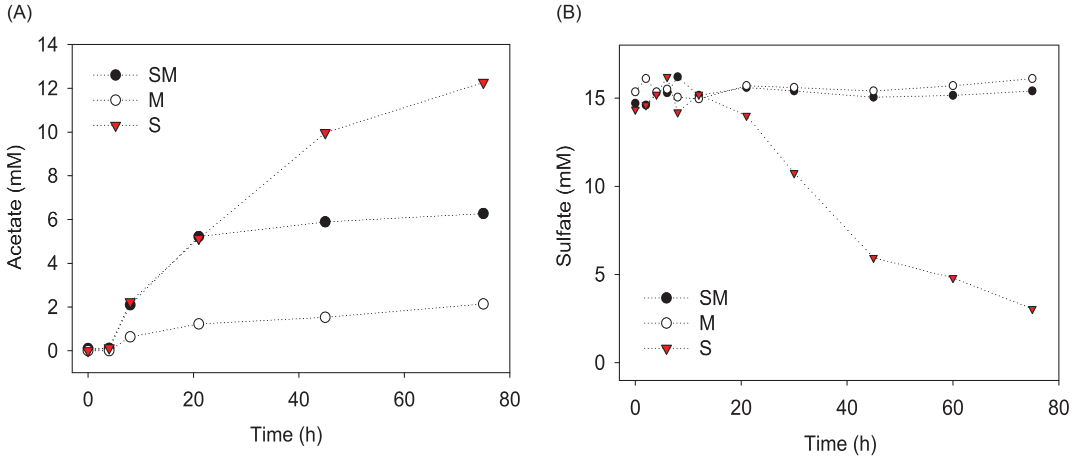

Supplementary Figure S3. Concentration of acetate **(A)** and sulfate **(B)** during anoxic incubation of pasteurized surface sediment slurries at 50°C. Slurries were amended with 2.5 g L^{-1} *Spirulina* powder (S), 10 mM molybdate (M) or both S and M (SM). Methane production was not detectable in any of the slurries during the incubation. The increased acetate accumulation observed in the S-slurry as compared to the SM-slurry is likely in part due to sulfate-dependent incomplete oxidation of organic fermentation products to acetate. The increased accumulation acetate in the S-slurry begins after 20 h of incubation as the sulfate concentration starts to decline.

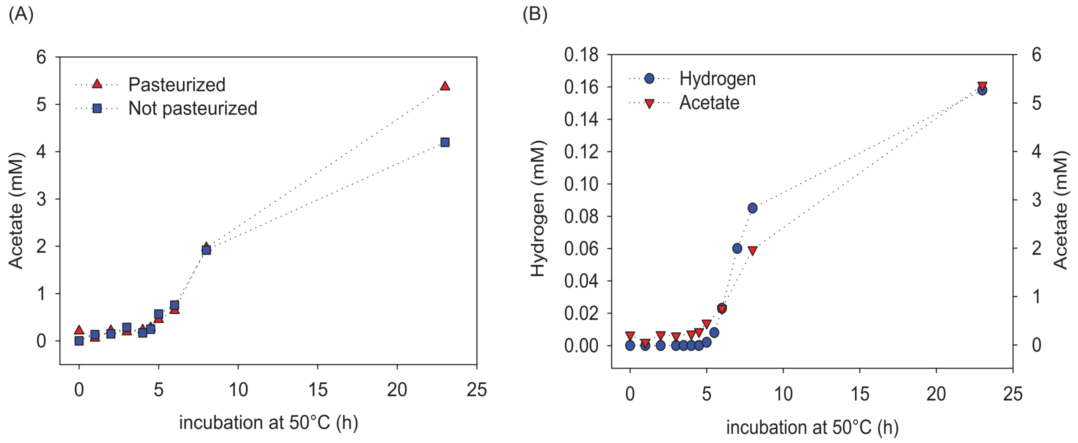

Supplementary Figure S4. Anoxic incubation at 50°C of surface sediment slurries amended with 2.5 g L^{-1} *Spirulina* powder and 10 mM molybdate. **(A)** Effect of pasteurization on acetate concentration. **(B)** Comparison of acetate and H_2 concentration in a pasteurized slurry.

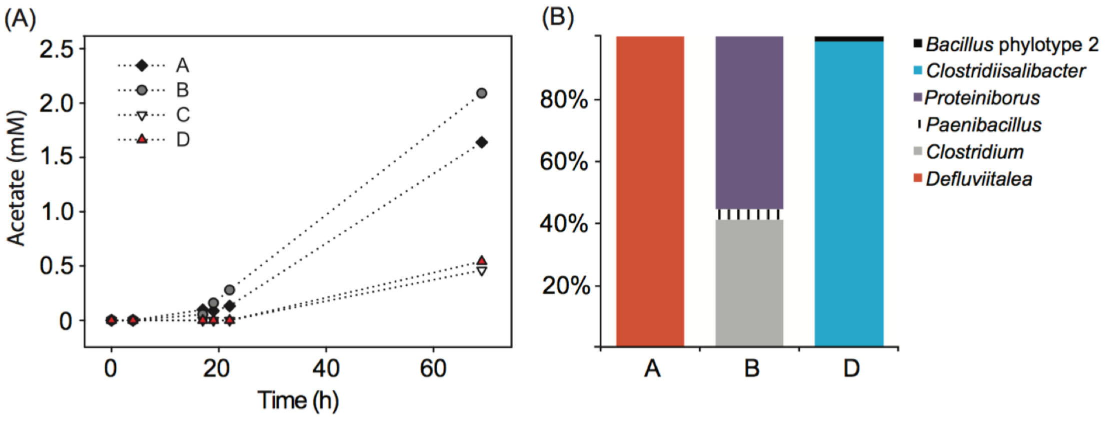

Supplementary Figure S5. **(A)** Concentration of acetate during anoxic incubation at 50°C of pasteurized planktonic cells from seawater. Cells were collected by filtering 4 L aliquots of seawater though 0.2 µm pore size membrane filter. Individual filters were placed in either sediment (A and B) or artificial medium (C and D) both amended with 0.25 g l^{-1} *Spirulina* powder and 10 mM molybdate, and immediately pasteurized and incubated. **(B)** 16S rRNA-based community composition in the incubations shown in panel A upon 17 h of incubation.

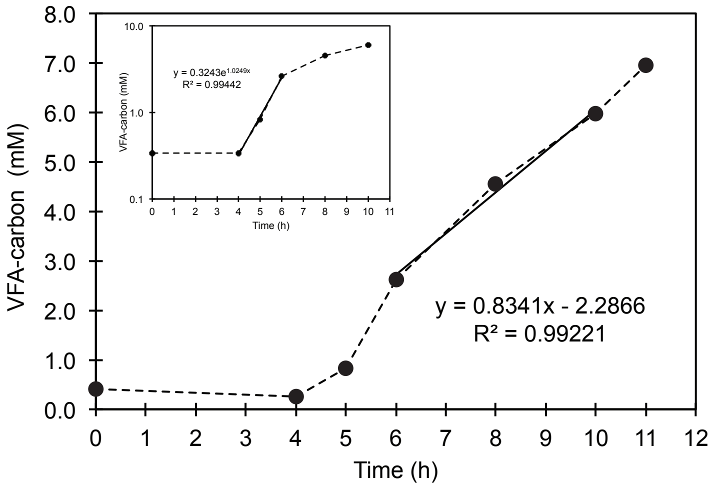

Supplementary Figure S6. VFA production by germinating endospores during anoxic incubation at 50°C of a pasteurized 1:9 (v/v) slurry of surface sediment amended with 2.5 g L^{-1} *Spirulina* extract and 10 mM molybdate. VFA-carbon was calculated by multiplying the molar amount of the VFAs shown in Figure 1A by the number of carbon atoms they contain. After 8 h of incubation the number of active cells was quantified by FISH. The VFA-carbon concentration increased linearly between 6 and 10 h of incubation at a rate of $8.3 \cdot 10^{-4}$ mol C h^{-1} l^{-1}. Upon 8 h of incubations $1.3 \cdot 10^{9}$ cells mL^{-1} were detectable by FISH corresponding to an average cell-specific VFA production rate of $6.4 \cdot 10^{-13}$ mol C $cell^{-1}$ h^{-1}. The number of TFEs in the slurry at the beginning of the exponential phase has been estimated by fitting an exponential curve between 4 and 6 hours. The initial rate of VFA-carbon production was calculated to be 0.332 mM h^{-1} (differentiating $y = 0.324\ e^{1.025x}$ and solving for x = 0). The insert shows the exponential fit on a logarithmic scale (where the average value of the first two measurements is used as zero) and its equation.

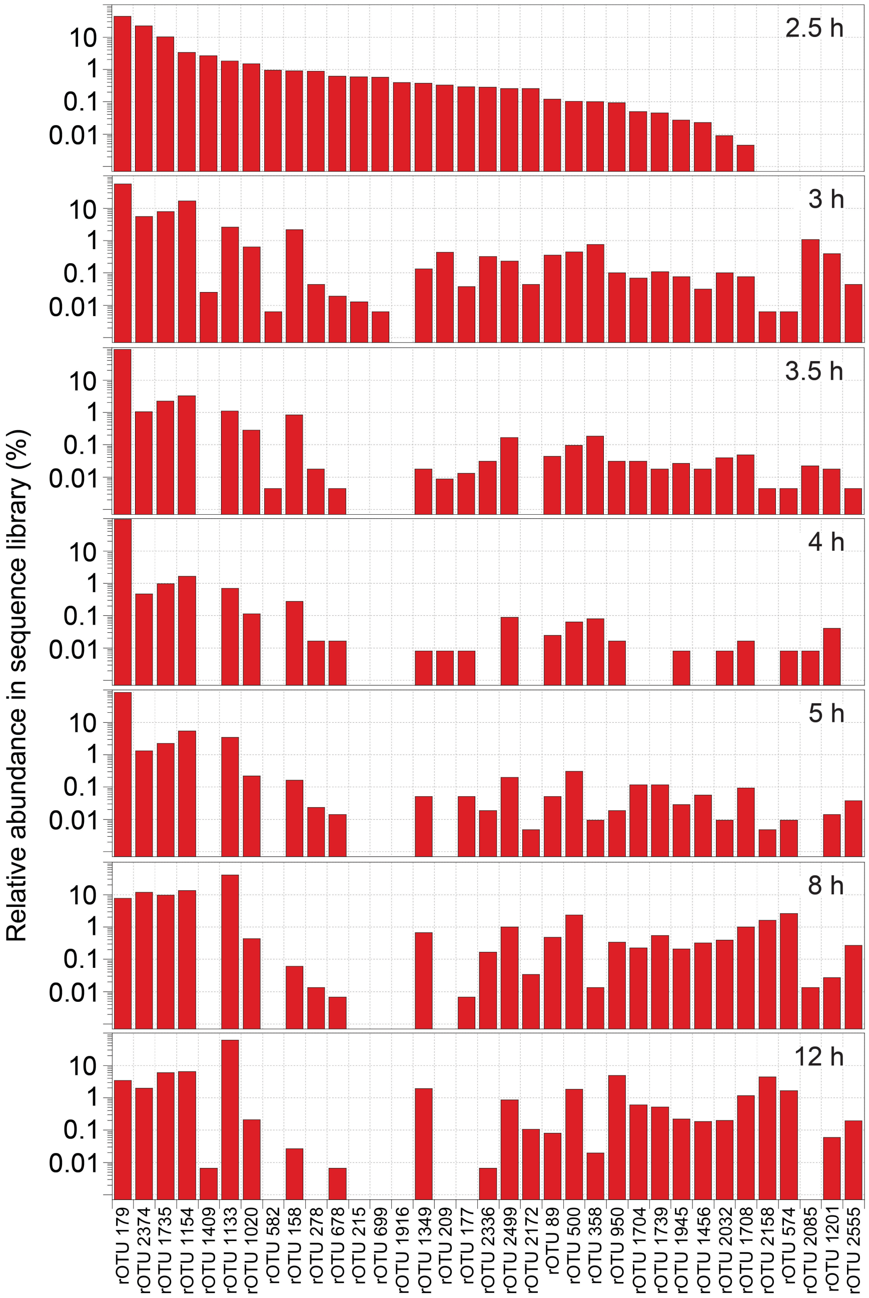

Supplementary Figure S7. Relative abundance of operational taxonomic units (OTUs) within 16S rRNA cDNA sequence libraries made from samples collected at different time points between 2.5 and 12 h of incubation of pasteurized sediment slurries incubated at 50°C under anoxic conditions. The relative abundance values were calculated by pooling data from two replicate libraries constructed for each time point (see Figure 1 in the main text of the manuscript). The relative abundance values are shown on a log scale on the Y-axes. The shown OTUs represent 92-98 % of the total number of sequences within the pooled libraries of the respective time points.

Supplementary Tables

Supplementary Table S1. Characteristics of cultured relatives of predominant TFEs

TFE OTU	Isolate§	Pairwise 16S rRNA sequence identity	Isolation source	Temperature range for growth (°C)	Reference
OTU358	*Bacillus thermolactis* [AY397764]	99%	Raw milk	40-60	(Coorevits et al., 2011)
OTU2374	*Defluviitalea saccharophila* [HQ020487]	99%	Wastewater treatment plant	40-60	(Jabari et al., 2012)
OTU1349	*Tepidimicrobium xylanilyticum* [EF522948]	96%	Anaerobic digester	25-67	(Niu et al., 2009)
OTU1133	*Caloranaerobacter* sp. TR13 [KC533829]	97%	Marine hydrothermal vent	37-75	(Zhou et al., 2015)
OTU179	*Tepidibacter formicigenes* [AY245527]	99%	Marine hydrothermal vent	35-55	(Urios et al., 2004)
OTU508	*Clostridium caminithermale* [AF458779]	97%	Marine hydrothermal vent	20-58	(Brisbarre et al., 2003)
OTU574	*Caminicella sporogenes* [NR_025485]	97%	Marine hydrotermal vent	45-65	(Alain et al., 2002)
OTU2172	*Brassicibacter thermophiles* [NR_137216]	99%	Marine sediment	30-70	(Wang et al., 2015)

§As identified by blastn search against the NCBI nr database. Genbank accession numbers are shown in brackets.

Supplementary Table S2. This table is available as a separate supplementary file.

frontiers

References

Alain, K., Pignet, P., Zbinden, M., Quillevere, M., Duchiron, F., Donval, J.-P., et al. (2002). *Caminicella sporogenes* gen. nov., sp. nov., a novel thermophilic spore-forming bacterium isolated from an East-Pacific Rise hydrothermal vent. *Int. J. Syst. Evol. Microbiol.* 52, 1621–1628. doi:10.1099/00207713-52-5-1621.

Brisbarre, N., Fardeau, M.-L., Cueff, V., Cayol, J.-L., Barbier, G., Cilia, V., et al. (2003). *Clostridium caminithermale* sp. nov., a slightly halophilic and moderately thermophilic bacterium isolated from an Atlantic deep-sea hydrothermal chimney. *Int. J. Syst. Evol. Microbiol.* 53, 1043–1049. doi:10.1099/ijs.0.02471-0.

Coorevits, A., Logan, N. A., Dinsdale, A. E., Halket, G., Scheldeman, P., Heyndrickx, M., et al. (2011). *Bacillus thermolactis* sp. nov., isolated from dairy farms, and emended description of *Bacillus thermoamylovorans*. *Int. J. Syst. Evol. Microbiol.* 61, 1954–1961. doi:10.1099/ijs.0.024240-0.

Jabari, L., Gannoun, H., Cayol, J.-L., Hamdi, M., Fauque, G., Ollivier, B., et al. (2012). Characterization of *Defluviitalea saccharophila* gen. nov., sp. nov., a thermophilic bacterium isolated from an upflow anaerobic filter treating abattoir wastewaters, and proposal of *Defluviitaleaceae* fam. nov. *Int. J. Syst. Evol. Microbiol.* 62, 550–5. doi:10.1099/ijs.0.030700-0.

Niu, L., Song, L., Liu, X., and Dong, X. (2009). *Tepidimicrobium xylanilyticum* sp. nov., an anaerobic xylanolytic bacterium, and emended description of the genus *Tepidimicrobium*. *Int. J. Syst. Evol. Microbiol.* 59, 2698–701. doi:10.1099/ijs.0.005124-0.

Urios, L., Cueff, V., Pignet, P., and Barbier, G. (2004). *Tepidibacter formicigenes* sp. nov., a novel spore-forming bacterium isolated from a Mid-Atlantic Ridge hydrothermal vent. *Int. J. Syst. Evol. Microbiol.* 54, 439–443. doi:10.1099/ijs.0.02836-0.

Wang, B., Li, F.-L., Tian, X.-X., Qu, L.-Y., and Ji, S.-Q. (2015). *Brassicibacter thermophilus* sp. nov., a thermophilic bacterium isolated from coastal sediment. *Int. J. Syst. Evol. Microbiol.* 65, 2870–2874. doi:10.1099/ijs.0.000348.

Zhou, M., Xie, Y., Dong, B., Liu, Q., and Chen, X. (2015). Draft genome sequence of *Caloranaerobacter* sp. TR13, an anaerobic thermophilic bacterium isolated from a deep-sea hydrothermal vent. *Genome Announc.* 3, e01491-15. doi:10.1128/genomeA.01491-15.
